# A78 BILIARY STENT USE IN ERCP: A QUALITY ASSURANCE STUDY ASSESSING ADHERENCE TO CLINICAL GUIDELINES AND COST OUTCOMES

**DOI:** 10.1093/jcag/gwac036.078

**Published:** 2023-03-07

**Authors:** A Decanini, S I S Alhaidari, I Alzahrani, M Alhanaee, M Mohamed, S Zepeda-Gomez, P Mathura, J Zhang, G Sandha

**Affiliations:** 1 Department of Medicine, University of Alberta, Edmonton, Canada; 2 Department of Medicine, Imam Abdulrahman bin Faisal University, Dammam, Saudi Arabia; 3 Alberta Health Services, Edmonton, Canada

## Abstract

**Background:**

A perceived increase in the use of biliary stents during endoscopic retrograde cholangiopancreatography (ERCP) was observed by our nursing team leader and brought to the attention of the Director of Endoscopy.

**Purpose:**

To assess a) biliary stent utilization during ERCP, b) the adherence for stent placement based on published guidelines, and c) the associated cost.

**Method:**

A chart review of all consecutive patients that underwent ERCP for one year (January 2020 to 2021) at the University of Alberta Hospital (UAH) was performed. The need for biliary stent placement was assessed independently by two blinded reviewers and compared with published guidelines. Costs were calculated using Alberta Health Services fee codes.

**Result(s):**

A total of 598 patients (316 F) with mean age of 60±19 years (range 3-99 years) underwent 842 ERCPs. Clinical indications for the initial ERCP were common bile duct (CBD) stones (376, 63%), malignant stricture (84, 14%), benign stricture (49, 8%), bile leak (27, 5%), stent removal (15, 3%), and others (47, 8%). Of the 244 patients that had a follow-up ERCP, the most common indications were stent removal (126, 52%), stent replacement (61, 25%), stent placement (28, 11%), and stone extraction (8, 3%). A total of 296 biliary stents were inserted, of which 223 stents (114 plastic, 109 metal) were inserted during the first ERCP (223/598, 37%) and 73 stents (43 plastic, 30 metal) during follow-up ERCP (73/244, 30%).

Of the 296 stents, 79 (27%) were inserted for indications not in accordance with published guidelines (63/223 initial ERCP, and 16/73 follow-up ERCP, *kappa*=0.62). Most of these were placed in CBD stone cases (61/63 initial ERCP, 6/16 follow-up ERCP). In the subgroup of 376 patients with CBD stones, 61 (16%) underwent stent placement not in accordance with published guidelines. The added cost of such stent insertions and follow-up ERCPs for stent removal was $130,000.

**Conclusion(s):**

Stent insertion not in accordance with published guidelines was identified in some patients with CBD stones presenting for ERCP. To reduce unnecessary follow-up procedures and healthcare resource utilization, ERCP stent insertion education based on published guidelines, as well as regular practice audit and feedback are required.

**Please acknowledge all funding agencies by checking the applicable boxes below:**

None

**Disclosure of Interest:**

None Declared

